# The Effect of Tuberculosis and HIV on the Epidemiological, Clinical, Virological, and Immunological Trajectory of COVID-19: Protocol for a Prospective Case-Ascertained and Surveillance Study

**DOI:** 10.2196/79438

**Published:** 2026-09-15

**Authors:** Azaria Diergaardt, Etuhole Iipumbu, Sibongile Netha, Pieter Steenkamp, Libertina Shiweva, Balladiah Kizito, Gunar Günther, Emmanuel Nepolo, Tadesse Woldetsadik, Stefan Niemann, Chawangwa Modongo, Mareli Claassens

**Affiliations:** 1 School of Medicine, University of Namibia, Bach Street, Windhoek, Namibia, +264 612063111; 2 Health Poverty Action, Gobabis, Namibia; 3 Victus Global Botswana Organization, Gabarone, Botswana; 4 Department of Pulmonology, Allergologie and Clinical Immunologie, Inselspital, Bern University Hospital, University of Bern, Bern, Switzerland; 5 Health Poverty Action, London, United Kingdom; 6 Molecular and Experimental Mycobacteriology Group, Research Center Borstel, Borstel, Germany; 7 Desmond Tutu Tuberculosis Centre, Department of Pediatrics and Child Health, Faculty of Medicine and Health Sciences, Stellenbosch University, Tygerberg, Western Cape, South Africa

**Keywords:** tuberculosis, HIV and AIDS, COVID-19, southern Africa, pandemics

## Abstract

**Background:**

The COVID-19 pandemic impacted over 620 million people, including approximately 9 million in Africa, and introduced uncertainties, especially regarding coinfection with tuberculosis (TB) or HIV. TB and HIV, both global epidemics, disproportionately affect low- and middle-income countries. Namibia and Botswana have reported high mortality rates due to TB and HIV, highlighting ongoing challenges.

**Objective:**

This paper outlines the protocol for a research study in Botswana and Namibia to address these uncertainties and enhance disease management for COVID-19, TB, and HIV, especially with coinfection. The central hypothesis is that TB or HIV coinfection increases the risk of adverse clinical, immunological, and virological COVID-19 outcomes.

**Methods:**

Core-NB is a prospective clinical cohort of patients with COVID-19 identified through facility-based surveillance. The objectives were to (1) characterize the early COVID-19 pandemic in conjunction with TB and HIV in clinical, epidemiological, virological, and immunological terms via a household transmission study; and (2) conduct targeted COVID-19 screening and testing in conjunction with TB and HIV case finding through a primary health care (PHC) facility surveillance study. For objective 1, participants were identified with reverse-transcription polymerase chain reaction (PCR) confirmation of COVID-19 at ministry of health–designated laboratories in each country. For objective 2, PHC attendees were enrolled independent of the reason for attending.

**Results:**

Index participants and consenting household contacts received home visits, including an initial enrollment visit on day 1 (the day of COVID-19 diagnosis for the index patient) and 3 follow-up visits taking place within 28 days of enrollment. Additionally, there were telephone follow-ups 3 months after enrollment. Age, sex, race, occupation, income, and educational attainment were recorded using a standardized questionnaire for all participants. Three respiratory specimens were obtained from each participant: (1) nasopharyngeal swab (SARS-CoV-2 RNA extraction and PCR); (2) sputum (GeneXpert Ultra active TB testing); and (3) extra sputum (an additional sputum sample was collected in case the GeneXpert Ultra test was positive to use for culture and drug susceptibility testing). The study was funded in October 2021, with data collection starting in June 2022 and finalized in February 2024. The household component enrolled 66 index patients with COVID-19 and 144 household contacts. In total, 1556 participants were enrolled in the PHC clinic component. The primary outcome data analyses are ongoing and expected to be published in July 2026, with secondary outcomes published in December 2026.

**Conclusions:**

This study will strengthen COVID-19, TB, and HIV diagnosis, surveillance, and control through (1) high-resolution surveillance and transmission data to establish guidelines and policies; (2) data to develop further interventions; (3) implementation of new technologies to improve care provision and boost the cooperation of stakeholders; (4) support for the development of skills and expertise in-country; and (5) contribution of evidence to build a regional network of expertise in the COVID-19, TB, and HIV coepidemics.

## Introduction

In December 2019, an emerging respiratory illness known as COVID-19 caused by SARS-CoV-2 originated in China and spread worldwide [1]. Recognizing its rapid worldwide transmission and impact, the World Health Organization (WHO) officially designated it as a pandemic on March 11, 2020. This pandemic affected over 620 million individuals across the globe, with an estimated 9 million cases reported in the African continent [2].

Emerging respiratory pathogens pose a significant challenge to public health systems worldwide, often characterized by initial uncertainty regarding their epidemiological, clinical, and virological characteristics. The COVID-19 pandemic exemplified this phenomenon, with many key parameters remaining uncertain during its early stages [2]. After its initial detection, COVID-19 had an unprecedented global impact, manifesting with a spectrum of clinical outcomes ranging from asymptomatic and mild cases to severe illness and death, particularly among older adults and individuals with underlying health conditions, including tuberculosis (TB) and HIV/AIDS [3,4]. However, a critical knowledge gap persisted concerning the influence of coinfections, notably HIV and TB, on both the susceptibility to acquiring COVID-19 and the outcomes of COVID-19 [5,6].

TB and HIV, two coexisting global epidemics, disproportionately affect low- and middle-income countries with fragile health care systems [7]. TB, caused by *Mycobacterium tuberculosis* complex strains, affects approximately 25% of the world’s population, resulting in more than 10 million new TB cases and 1.5 million deaths annually [7]. HIV continues to infect approximately 1.7 million individuals each year, causing an estimated 770,000 deaths, with particularly devastating effects in regions with high TB burdens [7], highlighting the ongoing challenges posed by the epidemic [8]. Given the overlapping prevalence of TB, HIV, and COVID-19 in countries such as Botswana and Namibia, where high prevalence rates of both TB and HIV exist [9,10], understanding the interplay among these diseases was crucial for effective management during the COVID-19 pandemic and remains important for managing all 3 epidemics. In Namibia, low rates of COVID-19 vaccination due to misinformation contributed to a high infection rate [10]. To our knowledge, no other studies investigating the coinfection of COVID-19, TB, and HIV have been conducted in Namibia and Botswana.

To address critical research gaps, studies investigating the impact of TB and HIV coinfections on COVID-19 outcomes are urgently needed. While some preliminary research has explored the relationship between TB coinfection and COVID-19 severity, such as a case-control study of 36 patients in China that showed a likely increase in susceptibility to SARS-CoV-2 infection and severity when infected with TB [11], larger, high-quality studies are necessary to provide comprehensive insights and develop evidence-based interventions to treat and prevent COVID-19 in countries with high TB and HIV burdens. From neighboring South Africa, with a similar TB and HIV burden, it is clear that the COVID-19 pandemic had a severe impact on TB and HIV services as well as morbidity and mortality [12-15]. A prospective observational study in ambulatory patients in Kenya, Uganda, and South Africa mirrored these findings [16].

This paper presents the protocol for a comprehensive research project designed to address these knowledge gaps by including household contact tracing and a clinical study within health care facilities. This initiative, focused on Botswana and Namibia, aimed to shed light on the complex dynamics of COVID-19, TB, and HIV coinfections and, ultimately, improve the management and prevention of these diseases in resource-constrained settings. The central hypothesis was that TB and HIV coinfection increase the risk of adverse clinical, immunological, and virological COVID-19 outcomes. The objectives were to (1) characterize the COVID-19 epidemic in conjunction with TB and HIV in terms of clinical, epidemiological, virological, and immunological data by conducting a household transmission study; and (2) conduct targeted COVID-19 screening and testing in conjunction with TB and HIV case finding through a primary health care (PHC) facility surveillance study.

## Methods

### Study Setting

The study was conducted in Namibia’s Omaheke region and in Gaborone, Botswana, from November 2021 to February 2024.

**Figure 1.**
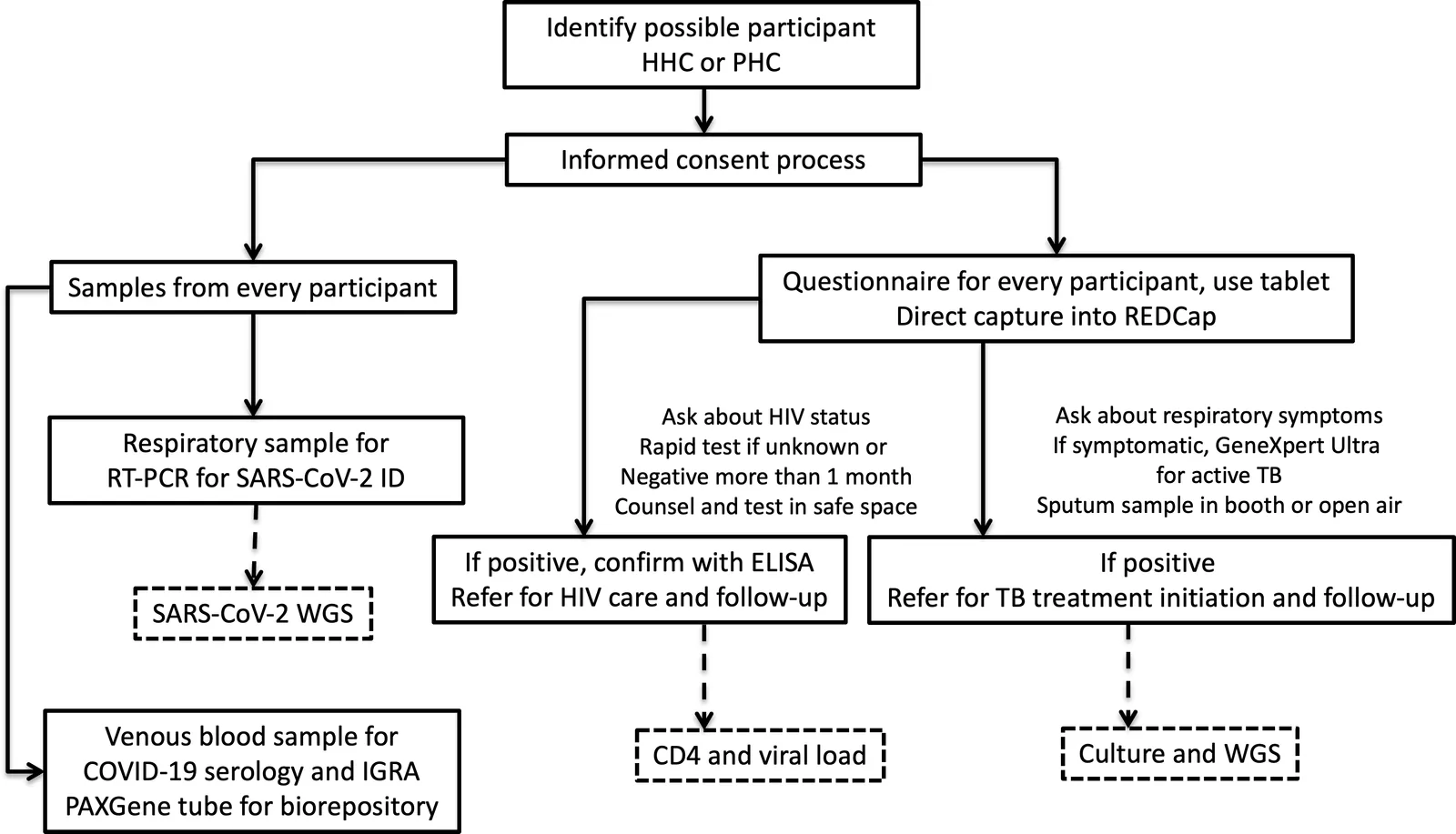
Core-NB study flow. ELISA: enzyme-linked immunosorbent assay; HHC: household contact; IGRA: interferon gamma release assay; PHC: primary health care; RT-PCR: reverse-transcription polymerase chain reaction; TB: tuberculosis; WGS: whole-genome sequencing.

PHC and household contact participants were identified and taken through the informed consent process (Figure 1). Questionnaires were administered directly using tablets, and samples from each participant were collected. Participants with unknown HIV status received a rapid test, and if it was positive, a viral load and CD4 test was conducted. Respiratory samples for reverse-transcription polymerase chain reaction (RT-PCR) for SARS-CoV-2 identification and GeneXpert Ultra (Cepheid) testing for TB identification were collected; when samples were positive, whole-genome sequencing (WGS) for *M tuberculosis* and a TB culture were conducted. Venous blood samples were collected for COVID-19 serology, an interferon gamma release assay (QIAreach QuantiFERON-TB; Qiagen GmbH) was used for TB infection and a PAXgene tube (BD Biosciences) was used for the biorepository.

### Patient and Public Involvement

Patients or the public were not involved in the design, conduct, reporting, or dissemination plans of our research. However, the national TB programs and the national and regional directorates of health were informed about the study and collaborated with the study teams on implementation. Community leaders and health care personnel were also informed about the study.

### Component 1: Household Contact Tracing

#### Design and Population

This was a prospective case-ascertained study of all identified confirmed patients with COVID-19 and their household contacts. The participants were identified based on laboratory RT-PCR confirmation of COVID-19 by the ministry of health’s designated laboratories in each country. Household contacts who agreed to participate in the study received 4 home visits, including an initial enrollment visit (day 1) on or as soon as possible after the day of COVID-19 diagnosis of the index patient and 3 follow-up visits that took place within 28 days of enrollment. Additionally, there were telephonic follow-ups 3 months after enrollment. During the visits, researchers collected specimens and gathered information regarding risk factors and symptoms from the primary patient with COVID-19 and from all individuals living in the same household. Information on age, sex, race, occupation, income, and educational attainment was collected using a standardized questionnaire for all participants.

#### Sample Size

The expected number of household contacts in Namibia and Botswana is 3 [17,18]. On the basis of a study involving influenza as a proxy (due to the limited availability of COVID-19 household transmission studies at the time of study design), a minimum sample size of 309 participants, including both index patients and contacts, was required to investigate household transmission of influenza-like illness [19,20]. Our goal was to include 360 participants to account for missing information, with an equal distribution between Namibia and Botswana. In the general adult population, the expected latent TB prevalence exceeds 30% [21], and HIV prevalence ranges from 11.5% [22] to 20.7% [23]. Consequently, we assumed that we would have included enough participants with latent TB and HIV to enable a meaningful comparison.

#### Data Collection Methods

This study involved several steps once a COVID-19 case was identified. A home visit was conducted to identify eligible household contacts, collect sociodemographic and clinical information, confirm secondary infections through molecular testing, and establish baseline antibody status. Data were captured directly into a REDCap web-based database using tablets, and specimens were collected from both the patient and their household contacts during home visits on days 1 (recruitment day), 7, 14, and 28. Household contacts completed a baseline questionnaire to monitor relevant symptoms and assess vaccine hesitancy. The final outcome data were collected 90 days after the initial visit.

#### Laboratory Evaluation

**Figure 2.**
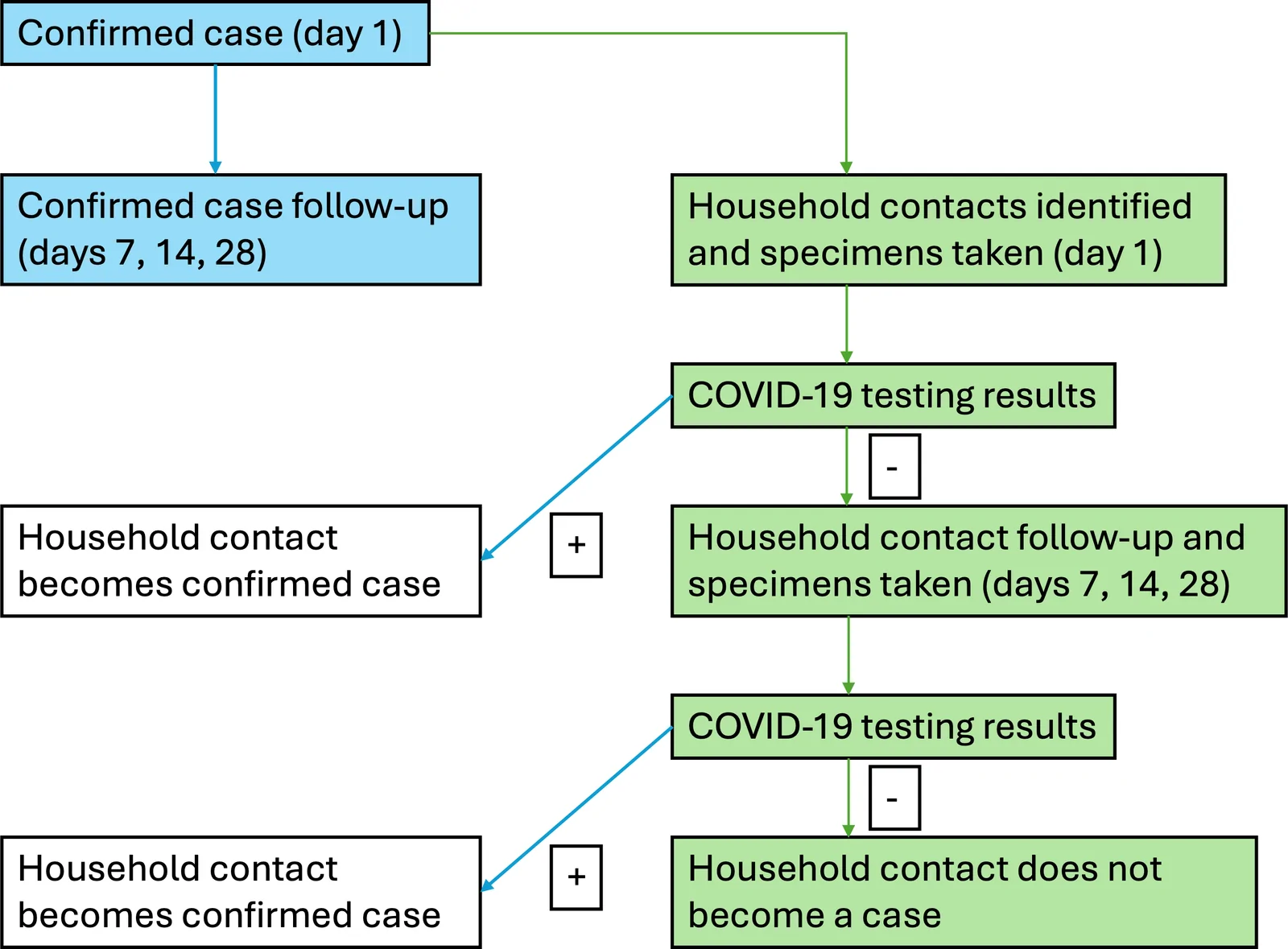
Case investigation algorithm and summary of data collection tools (household transmission study).

Upper respiratory tract specimens (nasopharyngeal and oropharyngeal swabs) and venous blood were collected from confirmed patients and their household contacts after the laboratory confirmation of the index patient. Collection took place during the initial home visit. Respiratory specimens were taken from all household members irrespective of symptoms, and the baseline questionnaire was administered. Blood samples were taken, stored in serum tubes, and subjected to the QIAreach QuantiFERON-TB assay testing for *M tuberculosis* infection. Additionally, for individuals with unknown HIV status, an HIV rapid test was conducted, with a confirmatory additional rapid test if it yielded a positive result. HIV-infected individuals underwent viral load and CD4 cell count testing. Participants were asked to provide a sputum sample to detect active TB via the GeneXpert Ultra. Follow-up specimens were collected as outlined in Figure 2. On day 7 and 14 visits, respiratory specimens were collected from household members for virological testing regardless of symptoms. On day 14 and 28 visits, a serum sample for SARS-CoV-Ab assay (Wantai BioPharm) and Platelia SARS-CoV-2 Total Ab (Bio-Rad) assay testing was obtained from household contacts. Extracted viral RNA and bacterial DNA were stored for subsequent viral targeted next-generation sequencing and bacterial WGS analysis, respectively. WGS was carried out at the Research Center Borstel (RCB), Borstel, Germany. A lung ultrasound was conducted on symptomatic index patients and household contacts, and a PAXgene (BD Biosciences) tube was collected for the sample repository on day 7.

Confirmed PHC cases were followed up on days 7, 14, and 28. The household contacts were identified, and specimens were collected for laboratory testing on day 1. If results were negative, the household contact was followed up on with specimen collections on days 7, 14, and 28. If the household contact tested positive for COVID-19, they became a confirmed case.

### Component 2: Surveillance Within Health Care Facilities

#### Study Design

Sentinel surveillance sites for COVID-19, TB, and HIV were established in 2 PHC facilities in each country. All attendees to the PHC facilities were approached consecutively, informed about the study, and invited to participate. Collection of specimens took place at baseline.

#### Sample Size

The determination of the sample size was based on a comparative analysis of prior studies conducted in Burkina Faso and South Africa [24,25]. Our assumption was rooted in the expectation that a proportion of PHC attendees would test positive for COVID-19, ranging from 5% to 20%. Given the uncertainty regarding the trajectory of the pandemic when the study was designed, we adopted conservative estimates.

To achieve a 5% CI width when measuring seroprevalence in the PHC study, we calculated that we would need to test 118 participants if the estimated seroprevalence was 1%, 337 participants for an estimated seroprevalence of 5%, and 595 participants for an estimated seroprevalence of 10%. Due to the uncertainty surrounding prevalence estimates, our target enrollment was set at 1190 participants, with an allowance for a 9% nonresponse rate. This resulted in a projected final sample size of 1120.

#### Data Collection Methods

##### Questionnaires

During the enrollment process, participants were interviewed by research assistants in a private area within the facility. Interviews were conducted in English or a local language based on the participants’ preference and typically lasted approximately 15 minutes (Multimedia Appendix 1). Participants were requested to provide consent for accessing their medical records, including national HIV and TB databases, to capture their clinical history and the clinical course of COVID-19 if relevant. Additionally, items addressing vaccine hesitancy were included in the questionnaire. HIV testing was conducted on individuals whose HIV status was unknown. HIV-infected patients who were not on treatment or newly diagnosed were referred to the facility for HIV counseling and care.

##### Specimens

Participants were directed to the study area for the collection of appropriate samples, including sputum or equivalent samples in children, nasopharyngeal or other respiratory samples, and blood specimens. Each specimen was labeled with a barcode and transported to the research laboratories for SARS-CoV-2 RT-PCR and GeneXpert Ultra testing for TB. Acknowledging the significance of asymptomatic and mild cases in propagating COVID-19 transmission, we implemented a policy of testing all consenting PHC attendees using RT-PCR irrespective of their presenting symptoms.

### Laboratory Evaluation

During the enrollment process, 3 respiratory specimens were collected from each participant:

- Nasopharyngeal swab—this specimen was obtained for SARS-CoV-2 RNA extraction and subsequent SARS-CoV-2 polymerase chain reaction testing.
- GeneXpert Ultra testing for TB—a specimen was collected for TB testing.
- Extra sputum sample—an additional sputum sample was collected in case the GeneXpert Ultra test was positive to use for culture and drug susceptibility testing.

Additionally, venous blood was drawn using standard phlebotomy techniques for immunological testing using the Wantai SARS-CoV-Ab and Platelia SARS-CoV-2 Total Ab assays, for the interferon gamma release assay to assess *M tuberculosis* infection, and for HIV CD4 and viral load testing in participants with HIV infection.

### Laboratory Methodology

This study involved several key laboratory procedures.

#### Detection of SARS-CoV-2

The presence of SARS-CoV-2 was identified in respiratory samples using the quantitative RT-PCR protocol established by the WHO [26].

#### Serological Testing

Assessment of immunological memory was carried out by measuring SARS-CoV-2–specific antibody levels through enzyme-linked immunosorbent assays. Plasma samples were used to determine the presence of immunoglobulin M and immunoglobulin G antibodies, which indicated recent (approximately 3-10 days) and past (>7 days) infection, respectively [27].

#### Sequencing

SARS-CoV-2 RNA was isolated from oro- and nasopharyngeal or gargled fluid specimens at the in-country laboratory. Positive samples underwent targeted next-generation sequencing to document the diversity of circulating SARS-CoV-2 genomes. GeneXpert Ultra positive sputum samples underwent a Mycobacteria Growth Indicator Tube (BD Biosciences) culture and WGS analysis. WGS was carried out at the RCB [28].

### Analysis

The PHC facility surveillance study and the household transmission study focused on the temporal, spatial, and demographic aspects of COVID-19 in conjunction with TB and HIV. It will provide insights into the clinical presentation and progression of COVID-19. Genomic analysis of SARS-CoV-2 specimens will provide a comprehensive understanding of the pandemic’s origins, the potential emergence of antiviral resistance mutations, and the identification of transmission chains. This analysis will involve comparison of the relatedness of virus isolates, which will aid in estimating the basic reproduction number. Serological testing will enable the quantification of events leading to immunological responses, whereas the analysis of PAXgene samples will yield insights into the nature of the host response. Additionally, lung ultrasound assessments will allow for the quantification of respiratory impairment attributable to COVID-19 stratified by severity, patient characteristics, and comorbidities. The primary outcome measures will be the (1) secondary attack rate of COVID-19 within household contacts and (2) coinfection rate of COVID-19, TB, and HIV in household contacts of confirmed patients with COVID-19. Secondary outcome measures will include descriptive statistics for risk factors and demographic data, as well as R0, incubation periods, and serial intervals, with regression analyses to investigate associations. For the qualitative interviews, a thematic analysis will be conducted.

### Ethical Considerations

#### Ethics Approval and Consent

Ethics approval was obtained from the University of Namibia Health Research Ethics Committee with number EXPC/01/21 and permission to conduct the study from the Ministry of Health and Social Services of Namibia with number 17/3/3/MC and from the Ministry of Health of Botswana with number HPDME 13/18/1. Well-trained staff experienced in the conduct of clinical research informed participants about the study. Written information was provided to potential participants, allowing them enough time to consider taking part and discuss it with their family or friends if desired. The consent forms were designed using plain language. For minors (individuals under the age of 18 years), consent was required to be signed by their parent or legal guardian prior to their inclusion. Additionally, assent forms were developed for children and adolescents aged 8 to 17 years who were deemed competent to provide their assent. All consent and assent forms contained detailed information regarding HIV testing and the potential secondary use of biological samples for future research purposes.

Data collection concerning individuals under 18 years of age was divided into two categories: (1) those who were of an appropriate age and possessed sufficient conceptual understanding to provide assent (typically aged around 8 years or older) and (2) those who were either too young or lacked the necessary conceptual understanding to assent. For the first category, we upheld their autonomy by requiring both written consent from their de facto caregiver (determined on the day of enrollment) and written assent from the child. In the case of the second category, only consent from their caregiver was required; however, field staff made appropriate efforts to verbally engage with the child and explain the purpose of data collection in the home. The field staff underwent specific training to assess the capacity of children to provide assent. It was emphasized that individuals had the right to decline participation in the study without providing a reason and, once enrolled, they retained the freedom to withdraw at any time without the obligation to provide an explanation. Potential participants were assured that their decision to take part or not would not impact the clinical care they would receive.

#### Minimizing the Risk of Study Participation

Given that the study was observational in design, there were no anticipated risks to participants in addition to routine tests conducted at the facility. Patients with comorbidities, particularly those with HIV or TB, were referred and followed up on to receive appropriate care through routine health services. Rapid HIV tests were conducted, and the results were promptly made available to participants. In the case of a confirmed positive result, participants were referred to the nearest health care facility for initiation of HIV care.

Respiratory sampling for both TB and COVID-19 is a simple, well-tolerated, and risk-free procedure that is routinely conducted in public health systems and known to be safe. The total blood volumes and blood volumes per visit were minimal and within the limits allowed for research. Lung ultrasound testing is a straightforward and risk-free procedure.

#### Privacy and Data Security

Case report forms, clinical notes, and administrative documentation were stored in a secure location, specifically in locked filing cabinets within a restricted-access room. These records will be retained for a period of 5 years following the conclusion of the study. Throughout this period, the data will remain accessible to competent or equivalent authorities and the funding entity provided suitable notice is given. Electronic data are securely stored in password-protected files on computers with password protection measures in place. All personally identifiable information is kept separate from clinical data and is solely linked through a personal identifier number. No personally identifiable information is transferred to any database used for data analysis. To ensure effective data management, the expertise of a skilled data officer was enlisted.

## Results

The Core-NB study was funded in October 2021, with data collection starting in June 2022 and finalized in February 2024. In the household component, 66 index patients with COVID-19 and 144 household contacts were enrolled. In total, 1556 participants were enrolled in the PHC clinic component. The primary outcome data analyses are ongoing and expected to be published in July 2026, with secondary outcomes published in December 2026.

## Discussion

Our study was developed in response to the COVID-19 global pandemic to answer critical questions concerning how this disease manifests itself in the context of low-resource countries with a high burden of poverty and in the presence of high rates of TB and HIV. We expect to find that TB and HIV coinfection increases the risk of adverse clinical, immunological, and virological COVID-19 outcomes, but these findings might be contextual and associated with other risk factors. In South Africa’s Western Cape province, people living with HIV have 2.75 times higher risk of mortality when infected with SARS-CoV-2, whereas those living with TB have 2.5 times higher risk. In addition, 52 of 100 people who die from COVID-19 have type 2 diabetes mellitus, 19 have hypertension, 12 have HIV, and 6 currently have or have previously had TB [29,30]. Our study results, to our knowledge, will be the first of their kind for the Namibian and Botswana context. As was done in South Africa, our study findings could be used to inform future efforts to combat the TB and HIV ongoing epidemics amid other infectious disease challenges [31,32].

The Core-NB study was conducted in 2 components. The first followed the WHO protocol for household transmission investigations in the context of COVID-19 [33], explored how commonly household transmission occurs, and described the spectrum of disease observed. We collected respiratory samples and blood to analyze viral molecular epidemiology and host immunological responses. The second component evaluated the presentation, diagnosis, and clinical characteristics of individuals presenting to sentinel health facilities in both countries for COVID-19, TB, and HIV. The study assisted in guiding the national response to COVID-19 in both countries, as well as assisting with our understanding of the pathogenesis of the virus in the context of TB and HIV, in turn providing vital information on how to deliver clinical care and how to design therapeutics and vaccines. The scientific advisory committee (SAC) organized for this study consisted of independent internationally renowned scientific experts. The SAC was invited to attend the kickoff meeting, as well as the progress and final meetings. They provided critical feedback after the scientific sessions. The European and Developing Countries Clinical Trials Partnership project officer was an observer on the SAC. After the meetings, the SAC submitted a feedback report to the consortium of partners who contributed to this study, including a concise strengths, weaknesses, opportunities, and threats analysis.

The strengths were (1) the very early stage of the COVID-19 pandemic, giving the opportunity to use WHO-proposed protocols to investigate virological and serological data, including the contribution of asymptomatic patients; (2) the high incidence of TB and HIV; (3) the established collaboration among the University of Namibia, Imperial College London, Victus Global Botswana Organization (VGBO), and RCB, including the ability to sequence viral RNA; (4) the expertise of the collaborators, including experience with TB and HIV research; (5) the 2-pronged strategy to capture early pandemic dynamics; (6) the buy-in of the ministries of health and other stakeholders; and (8) the possibility of sharing the technology regionally. The limitations were, first, the relatively little experience with COVID-19, which was mitigated by our genomic, epidemiological, microbiological, and local knowledge of both the TB and HIV epidemics and its applicability to other infectious diseases, as well as our ongoing collaborations with medical virologists and other experts. Second, it would have been preferable to conduct all analyses, including WGS, in country, but the establishment of a local next-generation sequencing platform is part of future projects. Third, viral RNA will be shipped to the RCB until the workflow has been implemented at the local laboratories, and there is an infection risk to the project staff, which needs to be mitigated through maximum risk reduction using personal protective equipment and hygiene rules, conscious handling of respiratory samples, and minimization of high-risk aerosol- and droplet-producing diagnostic and study-related procedures, such as sputum sampling.

This study will strengthen COVID-19, TB, and HIV diagnosis, surveillance, and control through (1) high-resolution surveillance and transmission data to establish guidelines and policies; (2) data to develop further interventions; (3) implementation of new technologies to improve care provision and boost the cooperation of stakeholders; (4) support for the development of skills and expertise in country; and (5) contribution of evidence to build a regional network of expertise in the COVID-19, TB, and HIV coepidemics.

In summary, this study worked toward establishing a regional clinical and research hub. This will provide training and capacity building for public health, laboratory techniques, and epidemiology in the future and assist with pandemic preparedness. This study developed a consortium of researchers who established research infrastructure, research training, data systems and laboratory systems and helped train the next generation of African researchers. Results were disseminated at international conferences, including the European and Developing Countries Clinical Trials Partnership forum and the European Society of Mycobacteriology annual conference. Publications will be shared with local ministries and community partners. Policy recommendations will be developed and disseminated once the primary outcome analyses are complete.

## Supplementary material

10.2196/79438 Multimedia Appendix 1 Questionnaire completed during interviews with participants of the Core-NB study.

10.2196/79438 Peer Review Report 1 Peer review report by the European and Developing Countries Clinical Trials Partnership.

## Abbreviations

PHC: primary health care
RCB: Research Center Borstel
RT-PCR: reverse-transcription polymerase chain reaction
SAC: scientific advisory committee
TB: tuberculosis
VGBO: Victus Global Botswana Organization
WGS: whole-genome sequencing
WHO: World Health Organization
