# Supplementary material for: The Effect of Tuberculosis and HIV on the Epidemiological, Clinical, Virological, and Immunological Trajectory of COVID-19: Protocol for a Prospective Case-Ascertained and Surveillance Study
Source: JMIR Res Protoc. 2026 Sep 15;15:e79438. doi: 10.2196/79438 (PMC13577614; doi:10.2196/79438)
Supplement: Supplementary file 1 [file resprot-v15-e79438-s002.pdf]

Consensus evaluation summary

**EDCTP Call Title:**	Mobilisation of funding for COVID-19 research in sub-Saharan Africa
**Grant Reference:**	RIA2020EF-2963
**Lead Applicant:**	Professor Stefan Niemann
**Coordinator:**	Forschungszentrum Borstel Leibniz-Zentrum fur Medizin und Biowissenschaften (FZB)
**Title:**	Collision of three global pandemics: the effect of tuberculosis and HIV on the epidemiological, clinical, virological and immunological trajectory of COVID-19 in Botswana and Namibia
During the Selection Committee meeting the assigned rapporteur must record the consensus evaluation summary from the selection committee.<br>Thereafter, the rapporteur and two other members of the committee must confirm their agreement of the below finalised consensus evaluation summary.<br>The below comments will be provided as feedback to the applicant.	

**Excellence** *(1000 word max)*
The proposal aims to study the effect of HIV and tuberculosis (TB) on the outcomes of COVID-19 disease as these two conditions impair the host immune response to other infectious disease. Given that there are currently few cases of COVID-19 diagnosed in both countries, the project will document the early stages of how the virus spreads within susceptible populations. The project includes two studies that will be conducted sequentially. The first study will follow the WHO protocol for household transmission investigations in the context of COVID-19. It will explore transmission frequency and describe the clinical spectrum of disease. Samples collected will also serve as basis for COVID-19 molecular epidemiology and host immunological response. The second study will evaluate the presentation, diagnosis and clinical characteristics of individuals presenting to sentinel health facilities in both countries. This proposal is within the scope of the call and aims to address a major gap in current knowledge that little is known regarding the effect of co-infections e.g. HIV and tuberculosis (TB), two coexisting global pandemics, on both the risk of acquiring COVID-19 and the outcome when of COVID-19 disease. TB and HIV disproportionately affect low and middle-income countries, with weak health systems and limited capacity to contain infectious disease outbreaks. High quality studies are urgently needed to improve the knowledge of the effect that TB and HIV may have on COVID-19 transmission and clinical, virological, and immunological outcomes. The study of TB co-infection among COVID-19 patients in China, found that those with interferon gamma release assay (IGRA) positive for Mycobacterium tuberculosis co- infection may have increased progression of severe COVID-19. Larger, high quality research studies are urgently needed to understand the impact of TB and HIV on COVID-19, and to develop evidence-based interventions to treat and prevent COVID-19 in countries with high TB and HIV prevalence. The study design will be a combination of Household contact tracing of all confirmed COVID- 19 cases and prospective clinical cohort studies based on surveillance of COVID-19 patients within healthcare facilities (a total of 1120 participants will be enrolled).Although the objectives are clear, the rational for sample size calculation was not presented if we want to study the effect of TB and HIV on the course of COVID-19 infection and severity. The need to support research activities in sub-Saharan Africa to manage and/or prevent the spread of the current COVID-19 outbreak is very well outlined in the proposal Collision of three global pandemics: the effect of tuberculosis and HIV on the epidemiological, clinical, virological and immunological trajectory of COVID-19 in Botswana and Namibia. The impending research proposal is addressing outstanding questions of how the virus spreads and how it causes pathology in resource limited settings with a high burden of poverty and with high rates

of TB and HIV. A particular gap in current knowledge is the effect of HIV and tuberculosis (TB) on the outcomes of COVID-19 disease as these two conditions impair the host immune response to other infectious disease. Understanding how these three pandemics interact is crucial for any intervention. The proposal explains well how the findings from this project will provide robust data to assist in guiding national responses to COVID-19 in both countries as well as assisting with our understanding of the pathogenesis of the virus in the context of TB and HIV, in turn providing vital information on how to deliver clinical care and how to design therapeutics and vaccines. The proposal is of paramount importance for countries with a high HIV prevalence and in many cases doubled with a co-infection of TB or DR-TB. Botswana is among those countries with the second highest HIV prevalence in the world and one of the highest TB prevalence rates. Namibia on the other hand is included for its high burden of TB. The household transmission study does not specify a prospective number of individuals that will be enrolled, however in the appendix doc it is said that a minimum sample of 309 persons should participate; it is further specified that all COVID 19 infected patients will be included. This is a bit confusing. Later in the proposal it is mentioned that 1120 participants will be enrolled for the primary healthcare facility (PHC) surveillance study. In the recruitment section it is said that once an infected patient is identified and enrolled a home visit is planned. The proposal does not mention how this is done. It might be that the household is not aware of a patients HIV or TB(co-) infection. Prof. Stefan Niemann is an experienced research lead for this proposal as he has led several studies using large scale WGS for analyzing resistance mechanism and spread of Mtb and other pathogens and collaborates with a diverse team from different disciplines and countries and institutions. The project proposal has well established teams in both countries with a long-lasting relationship with the MoH and TB programs. No mention of any qualitative approaches with HIV+ or TB infected patients experiences and perceptions and fears of a COVID 19 infection.

**Excellence score**	**4**

**Impact *(1000 word max)***
If properly conducted, the findings from this project will provide robust data to assist in guiding national responses to COVID-19 in both countries as well as assisting with understanding of the pathogenesis of the virus in the context of TB and HIV, in turn providing vital information on how to deliver clinical care and how to design therapeutics and vaccines. Given that there are few cases of COVID-19 currently diagnosed in Namibia and Botswana, the project will just document the early stages of how the virus spreads within susceptible populations. The authors will not then have the ability to test the hypothesis that TB and HIV will affect the severity of COVID-19 cases. The impact of the study will then be limited even if it will provide training and capacity building for public health, laboratory techniques and epidemiology. The expected impact of the research proposal is in line with the expected impacts listed for the call. The impact is well defined for different structural levels like personal and family to health care and national level in eight points. The study proposal outlines further on the potential of sub-studies including a qualitative analysis of the impact of the pandemic on the population. This project especially will be of direct benefit to individuals with COVID-19 and their families. The proposal will help to better understand how COVID 19 impacts individuals with and HIV or TB infection. The proposal is in line with the criteria set out by EDCTP to strengthen national and local research capacity, coordinate and collaborate with other activities in country, comply with international standards of good clinical practice and commit to open access and data sharing principles. The proposal is well thought of in sensitizing the community and giving health promotion sessions to explain the disease its transmission and to answer questions that might arise.

**Impact score**	**4**

**Implementation** *(1000 word max)*
The principal investigator is a well-recognized researcher and has a documented track records of previous grants. The team is composed of complementary researchers who are able to undertake the study, however as mentioned in the excellence section, as long as the objectives of the study will not be achieved through the design and sample size, the authors will not be able to demonstrate the effect of TB and HIV on the clinical course of COVID-19 cases. Five interlinked work packages will ensure the interaction between the three co-existing epidemics, COVID-19, TB and HIV, in Namibia and Botswana. Critical risks for implementation and proposed risk-mitigation measures were described but there was no mention on the number of COVID-19 cases needed to demonstrate an association between TB and HIV on the clinical

course of cases. The budget is justified and well balanced between partners. The proposed study is well defined and of specific importance in its approach bringing together three pandemics HIV TB and COVID 19.The project partners with the NGO Health Poverty Action, which has longstanding experience in community engagement and working especially with marginalized San communities in Namibia; drawing on their experience to assist in working with patient and community groups in both countries will certainly add to the studies quality and efficiency in implementation.<br>The proposed five work packages and included processes are described, explained and defined in detail and complement each other. A detailed ethical considerations plan and guideline in the annex of the proposal ensures that the study is compliant to all relevant ethical regulations. The institutions responsible for the different packages are well qualified and experienced to carry out the work successfully. The methods suggested are appropriate. Data collection and Data management is foreseen according to international standards. A detailed risk management plan is included.	
**Implementation score**	**4**
**Total score**	**12**
