# Supplementary material for: The Effect of Tuberculosis and HIV on the Epidemiological, Clinical, Virological, and Immunological Trajectory of COVID-19: Protocol for a Prospective Case-Ascertained and Surveillance Study
Source: JMIR Res Protoc. 2026 Sep 15;15:e79438. doi: 10.2196/79438 (PMC13577614; doi:10.2196/79438)
Supplement: Supplementary file 2 [file resprot-v15-e79438-s001.docx]

Form 1 – To be entered for Primary Case ( Day 1)

| Case ID |  |
| --- | --- |
| Date (dd/mm/yyyy) | _/ / _____ |

| **1. Case Identifier Information** | |
| --- | --- |
| First name |  |
| Surname |  |
| Sex | □ Male □ Female □ Other |
| Date of birth (dd/mm/yyyy) | _/ / ___  □ Unknown |
| Telephone (mobile) number |  |
| Age (years, months) | _ years months  □ Unknown |
| National ID/Passport Number (if applicable) |  |
| Country of residence |  |
| Nationality |  |
| Race | □ Black □ White □ Mixed □ Brown |
| Responsible health centre |  |
| Occupation | - Health worker - Work/stay home - Nursery/primary school/secondary school - Student - Other, specify: |
|  | For each occupation, specify location or facility: |

| **1b. Household information** | |
| --- | --- |
| Address Type | □ Current Address □ Past Address □ Other Address |
| Street Address/Ward |  |
| Plot Number |  |
| City/Town/Village |  |
| Latitude |  |
| Longitude |  |
| Household size (number of people who usually live in the  house, this will be varied depending on culture) |  |
| Number of rooms in house |  |
| Number of bedrooms |  |
| Age of each household member |  |

| **2. Epidemiology and Exposure** | |
| --- | --- |
| Case Classification | □ Confirmed □ Probable □ Suspect □ Not a case |
| Vaccine Status | □ Vaccinated □ Not Vaccinated |
| Vaccine Received  *(If Vaccinated)* | □ AstraZeneca □ Pfizer □ Jansen □ Sinovac |
| Travel | □ Abroad □ Different City/Town/Village |
| Date of first symptom onset (dd/mm/yyyy) | _/ / _  □ No symptoms □ Unknown |
| Date of first health facility visit (including traditional care) (dd/mm/yyyy) | _/ / _  □ Not applicable (na) □ Unknown |
| Total health facilities visited to date | □ na □ Unknown  Specify: |

| **3a. Primary case symptoms (from onset of symptoms)** *(If symptoms present)* | | |
| --- | --- | --- |
| Fever (≥38 °C) or history of fever | □ Yes □ No □ Unknown  If Yes, specify maximum temperature: °C | |
| **3b. Respiratory symptoms** *(If symptoms present)* | | |
| Sore throat | □ Yes □ No □ Unknown  If Yes, date (dd/mm/yyyy): _/ / _ | |
| Runny nose | □ Yes □ No □ Unknown | |
| Cough | □ Yes □ No □ Unknown  If Yes, date (dd/mm/yyyy): _/ / _ | |
| Shortness of breath | □ Yes □ No □ Unknown  If Yes, date (dd/mm/yyyy): _/ / _ | |
| Cough up blood | □ Yes □ No □ Unknown  If Yes, date (dd/mm/yyyy): _/ / _ | |
| **3c. Other symptoms** *(If symptoms present)* | | |
| Chills | □ Yes □ No □ Unknown | |
| Vomiting | □ Yes □ No □ Unknown | |
| Nausea | □ Yes □ No □ Unknown | |
| Diarrhoea | □ Yes □ No □ Unknown | |
| Headache | □ Yes □ No □ Unknown | |
| Rash | □ Yes □ No □ Unknown | |
| Conjunctivitis | □ Yes □ No □ Unknown | |
| Muscle aches | □ Yes □ No □ Unknown | |
| Joint ache | □ Yes □ No □ Unknown | |
| Loss of appetite | □ Yes □ No □ Unknown | |
| Loss of smell (anosmia) or taste | □ Yes □ No □ Unknown | |
| Nose bleed | □ Yes □ No □ Unknown | |
| Fatigue | □ Yes □ No □ Unknown | |
| Night sweats | □ Yes □ No □ Unknown  If Yes, date (dd/mm/yyyy): _/ / _ |  |
| Seizures | □ Yes □ No □ Unknown |  |
| Altered consciousness | □ Yes □ No □ Unknown |  |
| Other neurological signs | □ Yes □ No □ Unknown  If Yes, specify: |  |
| Other symptoms | □ Yes □ No □ Unknown  If Yes, specify: |  |

| **3. Primary case pre-existing condition(s)** | |
| --- | --- |
| Pregnancy  *(If Female)* | - Yes □ No □ Unknown   If Yes, specify trimester:   - First □ Second □ Third □ Unknown |
| Obesity | □ Yes □ No □ Unknown |
| Cancer | □ Yes □ No □ Unknown |
| Diabetes | □ Yes □ No □ Unknown |
| Immune deficiency disease | □ Yes □ No □ Unknown |
| HIV Status | □ Positive □ Negative □ Unknown  *(If unknown HIV tested to be conducted)* |
| Heart disease | □ Yes □ No □ Unknown |
| Asthma (requiring medication) | □ Yes □ No □ Unknown |
| Chronic lung disease (non-asthma) | □ Yes □ No □ Unknown |
| Chronic liver disease | □ Yes □ No □ Unknown |
| Chronic haematological disorder | □ Yes □ No □ Unknown |
| Chronic kidney disease | □ Yes □ No □ Unknown |
| Chronic neurological impairment/disease | □ Yes □ No □ Unknown |
| Organ or bone marrow recipient | □ Yes □ No □ Unknown |
| Other pre-existing condition(s) | □ Yes □ No □ Unknown  If Yes, specify: |

| **TB History** | |
| --- | --- |
| Have you ever had TB before? | □ Yes □ No □ Unknown |
| Previous TB Date | _/ / ___  □ Unknown |
| Was the TB treated? | □ Yes □ No □ Unknown |
| When did you take treatment? | _/ / ___  □ Unknown |
| What was the TB treatment outcome? | □ Cured □ Treatment Completed □ Defaulted |
| **HIV History** | |
| HIV Status | □ Positive □ Negative □ Discordant |
| When was the test conducted? | _/ / ___ |
| ART History | □ Patient has never taken ARTs □ Patient is taking ARTs □ Patient took ARTs but stopped |
| When were ARTs started | _/ / ___ |
| When were ARTs stopped | _/ / ___ |
| When were ARTs re-started | _/ / ___ |
| ART regimen | □ TLD □ ABC/3TC/DTG □ TAF-ED □ Atripla □ Other  If other specify |
| What is the current CD4 count |  |
| Date of CD4 count? | _/ / ___ |
| What is the current Viral Load? | < 400 <30 <25 0 Other  If other specify |
| Date of viral load? | _/ / ___ |

|  | **4. Form Completion** | |  |
| --- | --- | --- | --- |
|  | Name of data collector |  |  |
|  | Data collector institution |  |  |
|  | Form completion date (dd/mm/yyyy) | _/ / _ |  |
| **4b. Status of form completion** | | | |
| Form completed | | - Yes □ No or partially   If No or partially, reason:   - Missed - Not attempted - Not performed - Refusal - Other, specify: | |

*Form 2: To be entered for Contacts of confirmed cases (Day 1)*

| Case ID |  |
| --- | --- |
| Contact ID |  |
| Date (dd/mm/yyyy) | _/ / _____ |

| **1. Case Identifier Information** | |
| --- | --- |
| First name |  |
| Surname |  |
| Sex | □ Male □ Female □ Other |
| Date of birth (dd/mm/yyyy) | _/ / ___  □ Unknown |
| Telephone (mobile) number |  |
| Age (years, months) | _ years months  □ Unknown |
| National ID/Passport Number (if applicable) |  |
| Country of residence |  |
| Nationality |  |
| Race | □ Black □ White □ Mixed □ Brown |
| Responsible health centre |  |
| Occupation | - Health worker - Work/stay home - Nursery/primary school/secondary school - Student - Other, specify: |
|  | For each occupation, specify location or facility: |

| **1b. Household information** | |
| --- | --- |
| Address Type | □ Current Address □ Past Address □ Other Address |
| Date of last contact with the confirmed case (dd/mm/yyyy) | _/ / _ |
| Does the contact share a room (or usually share a room) with the primary case? | □ Yes □ No □ Unknown |
| Number of days during the time the case was ill at home that  were spent in contact with case (refer to household contact definition) |  |
| Did the contact take care of the case during the time he/she was ill at home before hospitalization? | □ Yes □ No □ Unknown |
| Did the contact hug the case during the time he/she was ill at  home before hospitalization? | □ Yes □ No □ Unknown |
| Did the contact kiss the case during the time he/she was ill at home before hospitalization? | □ Yes □ No □ Unknown |
| Did the contact shake hands with the case during the time  he/she was ill at home before hospitalization? | □ Yes □ No □ Unknown |
| Did the contact share a meal with the case during the time he/she was ill at home before hospitalization? | □ Yes □ No □ Unknown |
| Did the contact eat with hands from the same plate as the case during the time he/she was ill at home before  hospitalization? | □ Yes □ No □ Unknown |
| Did the contact share a drinking cup/glass with the case during the time he/she was ill at home before hospitalization? | □ Yes □ No □ Unknown |
| Did the contact share utensils with the case during the time  he/she was ill at home before hospitalization? | □ Yes □ No □ Unknown |
| Did the contact sleep in the same room as the case during the time he/she was ill at home before hospitalization? | □ Yes □ No □ Unknown |
| Did the contact share a toilet with the case during the time he/she was ill at home before hospitalization? | □ Yes □ No □ Unknown |

| **2. Epidemiology and Exposure** | |
| --- | --- |
| Case Classification | □ Confirmed □ Probable □ Suspect □ Not a case |
| Vaccine Status | □ Vaccinated □ Not Vaccinated |
| Vaccine Received  *(If Vaccinated)* | □ AstraZeneca □ Pfizer □ Jansen □ Sinovac |
| Travel | □ Abroad □ Different City/Town/Village |
| Date of first symptom onset (dd/mm/yyyy) | _/ / _  □ No symptoms □ Unknown |
| Date of first health facility visit (including traditional care) (dd/mm/yyyy) | _/ / _  □ Not applicable (na) □ Unknown |
| Total health facilities visited to date | □ na □ Unknown  Specify: |

| **3a. Primary case symptoms (from onset of symptoms)** *(If symptoms present)* | | | | |
| --- | --- | --- | --- | --- |
|  | Has the contact experienced any respiratory symptoms (sore throat, runny nose, cough, shortness of breath) in the period from 14 days **before** symptom onset in the confirmed case  until the present? | | - Yes - No   If No, skip to Section 5c | |
|  | Has the contact experienced any respiratory symptoms (sore throat, runny nose, cough, shortness of breath) in the period up to 14 days **after** the last contact or until the present date,  whichever is the earlier? | | - Yes - No | |
| Fever (≥38 °C) or history of fever | | □ Yes □ No □ Unknown  If Yes, specify maximum temperature: °C | | |
| **3b. Respiratory symptoms** *(If symptoms present)* | | | | |
| Sore throat | | □ Yes □ No □ Unknown  If Yes, date (dd/mm/yyyy): _/ / _ | | |
| Runny nose | | □ Yes □ No □ Unknown | | |
| Cough | | □ Yes □ No □ Unknown  If Yes, date (dd/mm/yyyy): _/ / _ | | |
| Shortness of breath | | □ Yes □ No □ Unknown  If Yes, date (dd/mm/yyyy): _/ / _ | | |
| **3c. Other symptoms** *(If symptoms present)* | | | | |
| Chills | | □ Yes □ No □ Unknown | | |
| Vomiting | | □ Yes □ No □ Unknown | | |
| Nausea | | □ Yes □ No □ Unknown | | |
| Diarrhoea | | □ Yes □ No □ Unknown | | |
| Headache | | □ Yes □ No □ Unknown | | |
| Rash | | □ Yes □ No □ Unknown | | |
| Conjunctivitis | | □ Yes □ No □ Unknown | | |
| Muscle aches | | □ Yes □ No □ Unknown | | |
| Joint ache | | □ Yes □ No □ Unknown | | |
| Loss of appetite | | □ Yes □ No □ Unknown | | |
| Loss of smell (anosmia) or taste | | □ Yes □ No □ Unknown | | |
| Nose bleed | | □ Yes □ No □ Unknown | | |
| Fatigue | | □ Yes □ No □ Unknown | | |
| Seizures | | □ Yes □ No □ Unknown | |  |
| Altered consciousness | | □ Yes □ No □ Unknown | |  |
| Other neurological signs | | □ Yes □ No □ Unknown  If Yes, specify: | |  |
| Other symptoms | | □ Yes □ No □ Unknown  If Yes, specify: | |  |

| **3. Primary case pre-existing condition(s)** | |
| --- | --- |
| Pregnancy  *(If Female)* | - Yes □ No □ Unknown   If Yes, specify trimester:   - First □ Second □ Third □ Unknown |
| Obesity | □ Yes □ No □ Unknown |
| Cancer | □ Yes □ No □ Unknown |
| Diabetes | □ Yes □ No □ Unknown |
| Immune deficiency disease | □ Yes □ No □ Unknown |
| HIV Status | □ Positive □ Negative □ Unknown |
| Heart disease | □ Yes □ No □ Unknown |
| Asthma (requiring medication) | □ Yes □ No □ Unknown |
| Chronic lung disease (non-asthma) | □ Yes □ No □ Unknown |
| Chronic liver disease | □ Yes □ No □ Unknown |
| Chronic haematological disorder | □ Yes □ No □ Unknown |
| Chronic kidney disease | □ Yes □ No □ Unknown |
| Chronic neurological impairment/disease | □ Yes □ No □ Unknown |
| Organ or bone marrow recipient | □ Yes □ No □ Unknown |
| Other pre-existing condition(s) | □ Yes □ No □ Unknown  If Yes, specify: |

|  | **4. Form Completion** | |  |
| --- | --- | --- | --- |
|  | Name of data collector |  |  |
|  | Data collector institution |  |  |
|  | Form completion date (dd/mm/yyyy) | _/ / _ |  |
| **4b. Status of form completion** | | | |
| Form completed | | - Yes □ No or partially   If No or partially, reason:   - Missed - Not attempted - Not performed - Refusal - Other, specify: | |

*Form 3: Follow Up Form. To be entered for Primary Case and Contacts of confirmed cases (Day 1, 7, 14, 28)*

| Case ID |  |
| --- | --- |
| Contact ID |  |
| Visit | □ Day 1 □ Day 7 □ Day 14 □ Day 28 |
| Date (dd/mm/yyyy) | _/ / _____ |

| **. Outcome** | |
| --- | --- |
| Outcome | □ Alive □ Dead □ N/A □ Unknown |
| Outcome current as of date (dd/mm/yyyy) | _/ / _  □ Unknown □ na |
| Hospitalization | - Yes □ No □ Unknown   If Yes, date of first hospitalization (dd/mm/yyyy)  _/ / _   - Unknown   If Yes, specify reason for hospitalization: |

|  | **4. Form Completion** | |  |
| --- | --- | --- | --- |
|  | Name of data collector |  |  |
|  | Data collector institution |  |  |
|  | Form completion date (dd/mm/yyyy) | _/ / _ |  |
| **4b. Status of form completion** | | | |
| Form completed | | - Yes □ No or partially   If No or partially, reason:   - Missed - Not attempted - Not performed - Refusal - Other, specify: | |

*Form 4: Sample Collection and Results Form – To be entered for Primary Case and Contacts of confirmed cases (Day 1, 7, 14, 28))*

| Case ID |  |
| --- | --- |
| Contact ID |  |
| Visit | □ Day 1 □ Day 7 □ Day 14 □ Day 28 |
| Date (dd/mm/yyyy) | _/ / _____ |

| **1a. Virology testing methods and results:** | |
| --- | --- |
| **Complete a form for each specimen collected and each type of test done:** | |
| **Sample ID** |  |
| **Date sample collected (dd/mm/yyyy)** | / _/ |
| **Date sample received (dd/mm/yyyy)** | / _/ |
| **Type of sample** | - Nasal swab - Throat swab   □ Nasopharyngeal swab  Other, specify: |
| **Type of test** | - PCR - Whole genome sequencing - Partial genome sequencing   Other, specify |
| **Result** | - POSITIVE for COVID-19 - NEGATIVE for COVID-19 - POSITIVE for other pathogens   Please specify which pathogens: |
| **Result date (dd/mm/yyyy)** | / / |
| **Specimens shipped**  **to other laboratory for confirmation** | - Yes   If Yes, specify date  _/ / _ If Yes, name of the laboratory:  No |

| **1b. Serology testing methods and results** | |
| --- | --- |
| **Complete a form for each specimen collected and each type of test done:** | |
| **Sample ID** |  |
| **Date sample collected (dd/mm/yyyy)** | / _/ |
| **Date sample received (dd/mm/yyyy)** | / _/ |
| **Type of sample** | - Serum - Other,   specify: |
| **Type of test** | Specify type (ELISA/IFA IgM/IgG,  neutralization assay, etc.): |
| **Result** | - POSITIVE   If positive, titre:   - NEGATIVE   INCONCLUSIVE |
| **Result date (dd/mm/yyyy)** | / / |
| **Specimens shipped**  **to other laboratory for confirmation** | - Yes   If Yes, specify date  _/ / _ If Yes, name of the laboratory:  No |

| **2. GeneXpert test and Results** | |
| --- | --- |
| **Complete a form for each specimen collected and each type of test done:** | |
| **Sample ID** |  |
| **Date sample collected (dd/mm/yyyy)** | / _/ |
| **Date sample received (dd/mm/yyyy)** | / _/ |
| **GeneXpert Result** | □ MTB Detected □ MTB Not Detected □, MTB Invalid/Error □, MTB Indeterminate □, MTB No Result |
| **RIF Resistance** | □ RIF Resistance Detected □ RIF Resistance Not Detected □ RIF Resistance Indeterminate |
| **MTB Detection** | □ Trace □ Low □ Medium □ High |

| **4. Form Completion** | |  |
| --- | --- | --- |
| Name of data collector |  |  |
| Data collector institution |  |  |
| Form completion date (dd/mm/yyyy) | _/ / _ |  |
| **4b. Status of form completion** | | |
| Form completed | - Yes □ No or partially   If No or partially, reason:   - Missed - Not attempted - Not performed - Refusal - Other, specify: | |

*Symptom diary for household contacts of confirmed cases (Day 1–28)*

| **Day** | **Symptoms*** | | | | | | |
| --- | --- | --- | --- | --- | --- | --- | --- |
|  | **No symptoms**  (check if none experienced) | **Fever**  ≥**38 °C** | **Runny nose** | **Cough** | **Sore throat** | **Shortness of breath** | **Other symptoms:**  **specify** |
| **0** | □ None | □ Yes □ No | □ Yes □ No | □ Yes □ No | □ Yes □ No | □ Yes □ No |  |
| **1** | □ None | □ Yes □ No | □ Yes □ No | □ Yes □ No | □ Yes □ No | □ Yes □ No |  |
| **2** | □ None | □ Yes □ No | □ Yes □ No | □ Yes □ No | □ Yes □ No | □ Yes □ No |  |
| **3** | □ None | □ Yes □ No | □ Yes □ No | □ Yes □ No | □ Yes □ No | □ Yes □ No |  |
| **4** | □ None | □ Yes □ No | □ Yes □ No | □ Yes □ No | □ Yes □ No | □ Yes □ No |  |
| **6** | □ None | □ Yes □ No | □ Yes □ No | □ Yes □ No | □ Yes □ No | □ Yes □ No |  |
| **7** | □ None | □ Yes □ No | □ Yes □ No | □ Yes □ No | □ Yes □ No | □ Yes □ No |  |
| **8** | □ None | □ Yes □ No | □ Yes □ No | □ Yes □ No | □ Yes □ No | □ Yes □ No |  |
| **9** | □ None | □ Yes □ No | □ Yes □ No | □ Yes □ No | □ Yes □ No | □ Yes □ No |  |
| **10** | □ None | □ Yes □ No | □ Yes □ No | □ Yes □ No | □ Yes □ No | □ Yes □ No |  |
| **11** | □ None | □ Yes □ No | □ Yes □ No | □ Yes □ No | □ Yes □ No | □ Yes □ No |  |
| **12** | □ None | □ Yes □ No | □ Yes □ No | □ Yes □ No | □ Yes □ No | □ Yes □ No |  |
| **13** | □ None | □ Yes □ No | □ Yes □ No | □ Yes □ No | □ Yes □ No | □ Yes □ No |  |
| **14** | □ None | □ Yes □ No | □ Yes □ No | □ Yes □ No | □ Yes □ No | □ Yes □ No |  |
| **…** |  |  |  |  |  |  |  |
| **28** | □ None | □ Yes □ No | □ Yes □ No | □ Yes □ No | □ Yes □ No | □ Yes □ No |  |

*Please select None for No symptoms. If no symptoms are experienced, then consider the entry complete.
